# Supplementary material for: Mapping the evolution of fertility support policies in China: A content and instrumental analysis
Source: PLoS One. 2025 Oct 9;20(10):e0332137. doi: 10.1371/journal.pone.0332137 (PMC12510515; doi:10.1371/journal.pone.0332137)
Supplement: S1 Appendix — (ZIP) [file pone.0332137.s001.zip › S1 Appendix. 226 original policy documents/74-国务院关于落实《政府工作报告》重点工作分工的意见(FBM-CLI-2-354051).doc]

国务院关于落实《政府工作报告》重点工作分工的意见

**发布部门：** [国务院](https://www.pkulaw.com/law/chl?Aggs.IssueDepartment=5)

**发文字号：**国发〔2021〕6号

**发布日期：**2021.03.19

**实施日期：**2021.03.19

**时效性：** [现行有效](https://www.pkulaw.com/law/chl?Aggs.TimelinessDic=01)

**效力级别：** [国务院规范性文件](https://www.pkulaw.com/law/chl?Aggs.EffectivenessDic=XC0203)

**法规类别：** [机关工作综合规定](https://www.pkulaw.com/law/chl?Aggs.Category=00301) [营商环境优化](https://dobusiness.pkulaw.com/)

国务院关于落实《政府工作报告》重点工作分工的意见
（国发〔2021〕6号）

各省、自治区、直辖市人民政府，国务院各部委、各直属机构：
　　为全面贯彻党的十九大和十九届二中、三中、四中、五中全会精神，深入落实中央经济工作会议精神和十三届全国人大四次会议通过的《政府工作报告》部署，做好今年政府工作，实现经济社会发展目标任务，现就《政府工作报告》确定的重点工作，提出分工意见如下：
　　一、落实2021年经济社会发展总体要求和主要预期目标
　　（一）总体要求。
　　**1．**在以习近平同志为核心的党中央坚强领导下，以习近平新时代中国特色社会主义思想为指导，全面贯彻党的十九大和十九届二中、三中、四中、五中全会精神，坚持稳中求进工作总基调，立足新发展阶段，贯彻新发展理念，构建新发展格局，以推动高质量发展为主题，以深化供给侧结构性改革为主线，以改革创新为根本动力，以满足人民日益增长的美好生活需要为根本目的，坚持系统观念，巩固拓展疫情防控和经济社会发展成果，更好统筹发展和安全，扎实做好“六稳”工作、全面落实“六保”任务，科学精准实施宏观政策，努力保持经济运行在合理区间，坚持扩大内需战略，强化科技战略支撑，扩大高水平对外开放，保持社会和谐稳定，确保“十四五”开好局起好步，以优异成绩庆祝中国共产党成立100周年。（国务院各部门分别负责，年内持续推进）
　　（二）今年发展主要预期目标。

　　**2．**坚定信心，攻坚克难，巩固恢复性增长基础，努力保持经济社会持续健康发展。国内生产总值增长6%以上；城镇新增就业1100万人以上，城镇调查失业率5.5%左右；居民消费价格涨幅3%左右；进出口量稳质升，国际收支基本平衡；居民收入稳步增长；生态环境质量进一步改善，单位国内生产总值能耗降低3%左右，主要污染物排放量继续下降；粮食产量保持在1.3万亿斤以上。（国家发展改革委、财政部、人力资源社会保障部、生态环境部、农业农村部、商务部、人民银行、海关总署、国家外汇局等按职责分工负责，12月底前完成）
　　（三）更好统筹疫情防控和经济社会发展。

　　**3．**坚持常态化防控和局部应急处置有机结合，继续毫不放松做好外防输入、内防反弹工作，抓好重点区域和关键环节防控，补上短板漏洞，严防出现聚集性疫情和散发病例传播扩散，有序推进疫苗研制和加快免费接种，提高科学精准防控能力和水平。（国务院各部门分别负责，年内持续推进）
　　二、保持宏观政策连续性稳定性可持续性，促进经济运行在合理区间
　　（四）在区间调控基础上加强定向调控、相机调控、精准调控。

　　**4．**宏观政策要继续为市场主体纾困，保持必要支持力度，不急转弯，根据形势变化适时调整完善，进一步巩固经济基本盘。科学把握国内经济运行态势，深入分析新情况新问题，及时实施政策预调微调，稳定市场预期。（国家发展改革委、财政部、人力资源社会保障部、人民银行等按职责分工负责，年内持续推进）
　　（五）积极的财政政策要提质增效、更可持续。

　　**5．**今年赤字率拟按3.2%左右安排、比去年有所下调，不再发行抗疫特别国债。财政支出总规模比去年增加，重点仍是加大对保就业保民生保市场主体的支持力度。中央本级支出继续安排负增长，进一步大幅压减非急需非刚性支出，对地方一般性转移支付增长7.8%、增幅明显高于去年，其中均衡性转移支付、县级基本财力保障机制奖补资金等增幅均超过10%。建立常态化财政资金直达机制并扩大范围，将2.8万亿元中央财政资金纳入直达机制、规模明显大于去年，为市县基层惠企利民提供更加及时有力的财力支持。各级政府都要节用为民、坚持过紧日子，确保基本民生支出只增不减，助力市场主体青山常在、生机盎然。（财政部牵头，国家发展改革委、人力资源社会保障部、人民银行等按职责分工负责，12月底前完成）
　　（六）优化和落实减税政策。

　　**6．**市场主体恢复元气、增强活力，需要再帮一把。继续执行制度性减税政策，延长小规模纳税人增值税优惠等部分阶段性政策执行期限，实施新的结构性减税举措，对冲部分政策调整带来的影响。将小规模纳税人增值税起征点从月销售额10万元提高到15万元。对小微企业和个体工商户年应纳税所得额不到100万元的部分，在现行优惠政策基础上，再减半征收所得税。各地要把减税政策及时落实到位，确保市场主体应享尽享。（财政部、税务总局牵头，4月底前出台相关政策，年内持续推进）
　　（七）稳健的货币政策要灵活精准、合理适度。

　　**7．**把服务实体经济放到更加突出的位置，处理好恢复经济与防范风险的关系。（人民银行、银保监会、证监会、国家发展改革委、财政部、国家外汇局等按职责分工负责，年内持续推进）货币供应量和社会融资规模增速与名义经济增速基本匹配，保持流动性合理充裕，保持宏观杠杆率基本稳定。（人民银行牵头，年内持续推进）保持人民币汇率在合理均衡水平上的基本稳定。（人民银行、国家外汇局牵头，年内持续推进）

　　**8．**进一步解决中小微企业融资难题。延续普惠小微企业贷款延期还本付息政策，加大再贷款再贴现支持普惠金融力度。（人民银行、银保监会、财政部、国家发展改革委、工业和信息化部等按职责分工负责，3月底前出台相关政策，年内持续推进）延长小微企业融资担保降费奖补政策，完善贷款风险分担补偿机制。（财政部牵头，银保监会等按职责分工负责，6月底前出台相关政策，年内持续推进）加快信用信息共享步伐。（国家发展改革委、人民银行牵头，年内持续推进）完善金融机构考核、评价和尽职免责制度。引导银行扩大信用贷款、持续增加首贷户，推广随借随还贷款，使资金更多流向科技创新、绿色发展，更多流向小微企业、个体工商户、新型农业经营主体，对受疫情持续影响行业企业给予定向支持。（人民银行、银保监会、财政部等按职责分工负责，6月底前出台相关政策，年内持续推进）大型商业银行普惠小微企业贷款增长30%以上。（银保监会、人民银行牵头，12月底前完成）创新供应链金融服务模式。（人民银行牵头，工业和信息化部、商务部、国务院国资委、市场监管总局、银保监会、国家外汇局等按职责分工负责，年内持续推进）适当降低小微企业支付手续费。（人民银行牵头，9月底前出台相关政策，年内持续推进）优化存款利率监管，推动实际贷款利率进一步降低，继续引导金融系统向实体经济让利。今年务必做到小微企业融资更便利、综合融资成本稳中有降。（人民银行、银保监会牵头，年内持续推进）
　　（八）就业优先政策要继续强化、聚力增效。

　　**9．**着力稳定现有岗位，对不裁员少裁员的企业，继续给予必要的财税、金融等政策支持。（人力资源社会保障部、财政部、税务总局、人民银行等按职责分工负责，年内持续推进）延续降低失业和工伤保险费率，扩大失业保险返还等阶段性稳岗政策惠及范围，延长以工代训政策实施期限。（人力资源社会保障部、财政部、税务总局牵头，4月底前出台相关政策，年内持续推进）拓宽市场化就业渠道，促进创业带动就业。（国家发展改革委、人力资源社会保障部牵头，年内持续推进）推动降低就业门槛，动态优化国家职业资格目录，降低或取消部分准入类职业资格考试工作年限要求。（人力资源社会保障部牵头，有关部门按职责分工负责，12月底前完成）支持和规范发展新就业形态，加快推进职业伤害保障试点。（人力资源社会保障部牵头，6月底前出台相关政策，年内持续推进）继续对灵活就业人员给予社保补贴。（人力资源社会保障部、财政部牵头，年内持续推进）推动放开在就业地参加社会保险的户籍限制。（人力资源社会保障部、国家医保局、财政部牵头，9月底前出台相关政策，年内持续推进）做好高校毕业生、退役军人、农民工等重点群体就业工作，完善残疾人、零就业家庭成员等困难人员就业帮扶政策，促进失业人员再就业。（人力资源社会保障部、国家发展改革委、教育部、财政部、农业农村部、退役军人部、中国残联等按职责分工负责，年内持续推进）

　　**10．**拓宽职业技能培训资金使用范围，开展大规模、多层次职业技能培训，完成职业技能提升和高职扩招三年行动目标，建设一批高技能人才培训基地。（人力资源社会保障部、国家发展改革委、教育部、财政部等按职责分工负责，年内持续推进）健全就业公共服务体系，实施提升就业服务质量工程。运用就业专项补助等资金，支持各类劳动力市场、人才市场、零工市场建设，广开就业门路，为有意愿有能力的人创造更多公平就业机会。（人力资源社会保障部牵头，6月底前出台相关政策，年内持续推进）
　　三、深入推进重点领域改革，更大激发市场主体活力
　　（九）进一步转变政府职能。

　　**11．**充分发挥市场在资源配置中的决定性作用，更好发挥政府作用，推动有效市场和有为政府更好结合。继续放宽市场准入，开展要素市场化配置综合改革试点，依法平等保护各类市场主体产权。（国家发展改革委牵头，年内持续推进）纵深推进“放管服”改革，加快营造市场化、法治化、国际化营商环境。将行政许可事项全部纳入清单管理。（国务院办公厅牵头，有关部门按职责分工负责，12月底前完成）深化“证照分离”改革，大力推进涉企审批减环节、减材料、减时限、减费用。（国务院办公厅、市场监管总局、司法部牵头，有关部门按职责分工负责，12月底前完成）完善市场主体退出机制，实行中小微企业简易注销制度。（市场监管总局牵头，税务总局等按职责分工负责，7月底前出台相关政策，年内持续推进）实施工业产品准入制度改革。（国务院办公厅、市场监管总局、司法部牵头，有关部门按职责分工负责，年内持续推进）推进汽车、电子电器等行业生产准入和流通管理全流程改革。（工业和信息化部、商务部、市场监管总局等按职责分工负责，12月底前完成）

　　**12．**把有效监管作为简政放权的必要保障，全面落实监管责任，加强对取消或下放审批事项的事中事后监管，完善分级分类监管政策，健全跨部门综合监管制度，提升监管能力，加大失信惩处力度，以公正监管促进优胜劣汰。（国务院办公厅、国家发展改革委、司法部、人民银行、市场监管总局牵头，有关部门按职责分工负责，适时出台相关政策，年内持续推进）大力推行“互联网＋监管”。建立健全政务数据共享协调机制，实现更多政务服务事项网上办、掌上办、一次办。（国务院办公厅牵头，有关部门按职责分工负责，年内持续推进）加强数字政府建设。（国务院办公厅牵头，国家发展改革委、财政部、科技部等按职责分工负责，10月底前出台相关政策，年内持续推进）推动电子证照扩大应用领域和全国互通互认。（国务院办公厅牵头，有关部门按职责分工负责，11月底前出台相关政策，年内持续推进）企业和群众经常办理的事项，今年要基本实现“跨省通办”。（国务院办公厅牵头，公安部、民政部、司法部、人力资源社会保障部、自然资源部、住房城乡建设部、交通运输部、国家卫生健康委、市场监管总局、国家医保局、国家林草局、国家邮政局、中国残联等按职责分工负责，12月底前完成当年任务）
　　（十）用改革办法推动降低企业生产经营成本。

　　**13．**推进能源、交通、电信等基础性行业改革，提高服务效率，降低收费水平。（国家发展改革委、工业和信息化部、交通运输部、国家能源局、国家铁路局、中国民航局、国家邮政局、中国国家铁路集团有限公司等按职责分工负责，年内持续推进）允许所有制造业企业参与电力市场化交易，进一步清理用电不合理加价，继续推动降低一般工商业电价。（国家发展改革委、国家能源局、财政部、市场监管总局等按职责分工负责，12月底前完成）中小企业宽带和专线平均资费再降10%。（工业和信息化部牵头，12月底前完成）全面推广高速公路差异化收费，坚决整治违规设置妨碍货车通行的道路限高限宽设施和检查卡点。（交通运输部、国家发展改革委、财政部、公安部、生态环境部、住房城乡建设部等按职责分工负责，12月底前完成）取消港口建设费，将民航发展基金航空公司征收标准降低20%。（财政部、交通运输部、中国民航局等按职责分工负责，6月底前出台相关政策，年内持续推进）鼓励受疫情影响较大的地方对承租国有房屋的服务业小微企业和个体工商户减免租金。（住房城乡建设部、国家发展改革委、财政部、国务院国资委、市场监管总局等按职责分工负责，年内持续推进）

　　**14．**推动各类中介机构公开服务条件、流程、时限和收费标准。（国家发展改革委、市场监管总局等按职责分工负责，年内持续推进）要严控非税收入不合理增长，严厉整治乱收费、乱罚款、乱摊派，不得扰民渔利，让市场主体安心经营、轻装前行。（财政部、市场监管总局、国家发展改革委、税务总局、民政部、工业和信息化部、公安部、应急部等按职责分工负责，年内持续推进）
　　（十一）促进多种所有制经济共同发展。

　　**15．**坚持和完善社会主义基本经济制度。毫不动摇巩固和发展公有制经济，毫不动摇鼓励、支持、引导非公有制经济发展。各类市场主体都是国家现代化的建设者，要一视同仁、平等对待。（国家发展改革委、工业和信息化部、全国工商联等按职责分工负责，年内持续推进）深入实施国企改革三年行动，做强做优做大国有资本和国有企业。（国务院国资委牵头，年内持续推进）深化国有企业混合所有制改革。（国家发展改革委、国务院国资委牵头，年内持续推进）构建亲清政商关系，破除制约民营企业发展的各种壁垒。弘扬企业家精神。（国家发展改革委、工业和信息化部、国务院国资委、全国工商联等按职责分工负责，年内持续推进）健全防范和化解拖欠中小企业账款长效机制。（工业和信息化部、财政部、国务院国资委牵头，年内持续推进）国家支持平台企业创新发展、增强国际竞争力，同时要依法规范发展，健全数字规则。强化反垄断和防止资本无序扩张，坚决维护公平竞争市场环境。（国家发展改革委、科技部、工业和信息化部、财政部、商务部、人民银行、市场监管总局、全国工商联等按职责分工负责，9月底前出台相关政策，年内持续推进）
　　（十二）深化财税金融体制改革。

　　**16．**强化预算约束和绩效管理，加大预算公开力度，精简享受税费优惠政策的办理流程和手续。落实中央与地方财政事权和支出责任划分改革方案。（财政部牵头，税务总局等按职责分工负责，年内持续推进）健全地方税体系。（财政部、税务总局牵头，年内持续推进）继续多渠道补充中小银行资本、强化公司治理。（银保监会、财政部、人民银行等按职责分工负责，6月底前出台相关政策，年内持续推进）深化农村信用社改革。（银保监会牵头，人民银行、财政部、农业农村部等按职责分工负责，年内持续推进）推进政策性银行分类分账改革。（财政部、人民银行、银保监会等按职责分工负责，12月底前出台相关政策）提升保险保障和服务功能。（银保监会牵头，年内持续推进）稳步推进注册制改革，完善常态化退市机制，加强债券市场建设，更好发挥多层次资本市场作用，拓展市场主体融资渠道。（证监会、人民银行、国家发展改革委等按职责分工负责，年内持续推进）强化金融控股公司和金融科技监管，确保金融创新在审慎监管的前提下进行。完善金融风险处置工作机制，压实各方责任，坚决守住不发生系统性风险的底线。（人民银行、银保监会、证监会、国家外汇局、国家发展改革委、财政部、国务院国资委等按职责分工负责，年内持续推进）
　　四、依靠创新推动实体经济高质量发展，培育壮大新动能
　　（十三）提升科技创新能力。

　　**17．**强化国家战略科技力量，推进国家实验室建设，完善科技项目和创新基地布局。实施好关键核心技术攻关工程，深入谋划推进“科技创新2030-重大项目”，改革科技重大专项实施方式，推广“揭榜挂帅”等机制。（科技部、国家发展改革委牵头，财政部、工业和信息化部、教育部、人力资源社会保障部、国务院国资委、中科院、工程院、国家国防科工局、国家自然科学基金委等按职责分工负责，年内持续推进）支持有条件的地方建设国际和区域科技创新中心，增强国家自主创新示范区等带动作用。（国家发展改革委、科技部牵头，年内持续推进）发展疾病防治攻关等民生科技。（科技部、国家发展改革委、国家卫生健康委牵头，有关部门按职责分工负责，年内持续推进）促进科技开放合作。（科技部、外交部、国家发展改革委、中科院、工程院、国家自然科学基金委等按职责分工负责，年内持续推进）加强知识产权保护。（国家知识产权局牵头，年内持续推进）加强科研诚信建设，弘扬科学精神，营造良好创新生态。（科技部牵头，教育部、人力资源社会保障部、中科院、工程院、中国科协、国家自然科学基金委等按职责分工负责，年内持续推进）基础研究是科技创新的源头，要健全稳定支持机制，大幅增加投入，中央本级基础研究支出增长10.6%，落实扩大经费使用自主权政策，优化项目申报、评审、经费管理、人才评价和激励机制，努力消除科研人员不合理负担，使他们能够沉下心来致力科学探索，以“十年磨一剑”精神在关键核心领域实现重大突破。（财政部、科技部、教育部、人力资源社会保障部、国家国防科工局、中科院、工程院、国家自然科学基金委等按职责分工负责，年内持续推进）
　　（十四）运用市场化机制激励企业创新。

　　**18．**促进科技创新与实体经济深度融合，更好发挥创新驱动发展作用。强化企业创新主体地位，鼓励领军企业组建创新联合体，拓展产学研用融合通道，健全科技成果产权激励机制，完善创业投资监管体制和发展政策。（国家发展改革委、科技部、财政部、工业和信息化部、国务院国资委、人民银行、证监会、银保监会、全国工商联等按职责分工负责，年内持续推进）纵深推进大众创业万众创新。（国家发展改革委牵头，9月底前出台相关政策，年内持续推进）延续执行企业研发费用加计扣除75%政策，将制造业企业加计扣除比例提高到100%，用税收优惠机制激励企业加大研发投入，着力推动企业以创新引领发展。（财政部、税务总局、科技部牵头，4月底前出台相关政策，年内持续推进）
　　（十五）优化和稳定产业链供应链。

　　**19．**继续完成“三去一降一补”重要任务。（国家发展改革委、工业和信息化部、财政部、住房城乡建设部、人民银行、国务院国资委等按职责分工负责，年内持续推进）对先进制造业企业按月全额退还增值税增量留抵税额。（财政部、税务总局牵头，6月底前出台相关政策，年内持续推进）提高制造业贷款比重，扩大制造业设备更新和技术改造投资。（国家发展改革委、工业和信息化部、财政部、人民银行、银保监会等按职责分工负责，年内持续推进）增强产业链供应链自主可控能力。（国家发展改革委、工业和信息化部、科技部、国务院国资委、国家国防科工局等按职责分工负责，年内持续推进）实施好产业基础再造工程，发挥大企业引领支撑和中小微企业协作配套作用。（工业和信息化部牵头，国家发展改革委、科技部、财政部、国务院国资委等按职责分工负责，9月底前出台相关政策，年内持续推进）发展工业互联网，促进产业链和创新链融合，搭建更多共性技术研发平台，提升中小微企业创新能力和专业化水平。（工业和信息化部牵头，科技部等按职责分工负责，7月底前出台相关政策，年内持续推进）加大5G网络和千兆光网建设力度，丰富应用场景。（工业和信息化部、国家发展改革委牵头，9月底前出台相关政策，年内持续推进）加强网络安全、数据安全和个人信息保护。（国家发展改革委、科技部、工业和信息化部、公安部、安全部、司法部、市场监管总局等按职责分工负责，年内持续推进）统筹新兴产业布局。（国家发展改革委牵头，年内持续推进）加强质量基础设施建设，深入实施质量提升行动，完善标准体系，促进产业链上下游标准有效衔接，弘扬工匠精神，以精工细作提升中国制造品质。（市场监管总局牵头，国家发展改革委、农业农村部、科技部、教育部、工业和信息化部、财政部、人力资源社会保障部、交通运输部、商务部、国务院国资委、国家国防科工局、国家知识产权局、全国总工会等按职责分工负责，年内持续推进）
　　五、坚持扩大内需这个战略基点，充分挖掘国内市场潜力
　　（十六）稳定和扩大消费。

　　**20．**紧紧围绕改善民生拓展需求，促进消费与投资有效结合，实现供需更高水平动态平衡。（国家发展改革委牵头，年内持续推进）多渠道增加居民收入。（国家发展改革委、财政部、人力资源社会保障部等按职责分工负责，年内持续推进）健全城乡流通体系，加快电商、快递进农村，扩大县乡消费。（商务部、国家发展改革委、财政部、农业农村部、交通运输部、国家邮政局、供销合作总社等按职责分工负责，年内持续推进）稳定增加汽车、家电等大宗消费，取消对二手车交易不合理限制，增加停车场、充电桩、换电站等设施，加快建设动力电池回收利用体系。（国家发展改革委、工业和信息化部、财政部、生态环境部、住房城乡建设部、交通运输部、商务部、国家能源局等按职责分工负责，年内持续推进）发展健康、文化、旅游、体育等服务消费。鼓励企业创新产品和服务，便利新产品市场准入，推进内外贸产品同线同标同质。保障小店商铺等便民服务业有序运营。运用好“互联网＋”，推进线上线下更广更深融合，发展新业态新模式，为消费者提供更多便捷舒心的服务和产品。引导平台企业合理降低商户服务费。（国家发展改革委、教育部、科技部、工业和信息化部、民政部、财政部、住房城乡建设部、农业农村部、商务部、文化和旅游部、市场监管总局、体育总局、国家医保局、国家邮政局、国家中医药局、国家药监局等按职责分工负责，年内持续推进）稳步提高消费能力，改善消费环境，让居民能消费、愿消费，以促进民生改善和经济发展。（国家发展改革委、民政部、人力资源社会保障部、农业农村部、商务部、市场监管总局、国家医保局等按职责分工负责，年内持续推进）
　　（十七）扩大有效投资。

　　**21．**今年拟安排地方政府专项债券3.65万亿元，优化债券资金使用，优先支持在建工程，合理扩大使用范围。中央预算内投资安排6100亿元。（国家发展改革委、财政部等按职责分工负责，年内持续推进）继续支持促进区域协调发展的重大工程，推进“两新一重”建设，实施一批交通、能源、水利等重大工程项目，建设信息网络等新型基础设施，发展现代物流体系。政府投资更多向惠及面广的民生项目倾斜，新开工改造城镇老旧小区5.3万个，提升县城公共服务水平。（国家发展改革委、财政部、工业和信息化部、生态环境部、住房城乡建设部、交通运输部、水利部、农业农村部、民政部、商务部、国家卫生健康委、国家能源局、中国民航局、国家铁路局、国家邮政局、国家乡村振兴局、中国国家铁路集团有限公司等按职责分工负责，年内持续推进）简化投资审批程序，推进实施企业投资项目承诺制。深化工程建设项目审批制度改革。完善支持社会资本参与政策，进一步拆除妨碍民间投资的各种藩篱，在更多领域让社会资本进得来、能发展、有作为。（国家发展改革委、财政部、自然资源部、生态环境部、住房城乡建设部、水利部、人民银行、银保监会等按职责分工负责，年内持续推进）
　　六、全面实施乡村振兴战略，促进农业稳定发展和农民增收
　　（十八）做好巩固拓展脱贫攻坚成果同乡村振兴有效衔接。

　　**22．**接续推进脱贫地区发展，对脱贫县从脱贫之日起设立5年过渡期，保持主要帮扶政策总体稳定。（国家乡村振兴局牵头，年内持续推进）健全防止返贫动态监测和帮扶机制，促进脱贫人口稳定就业，加大技能培训力度，发展壮大脱贫地区产业，做好易地搬迁后续扶持，分层分类加强对农村低收入人口常态化帮扶，确保不发生规模性返贫。（国家乡村振兴局、国家发展改革委、民政部、住房城乡建设部、人力资源社会保障部、农业农村部、商务部、文化和旅游部、供销合作总社、人民银行、银保监会等按职责分工负责，年内持续推进）在西部地区脱贫县中集中支持一批乡村振兴重点帮扶县。（国家乡村振兴局牵头，年内持续推进）坚持和完善东西部协作和对口支援机制，发挥中央单位和社会力量帮扶作用，继续支持脱贫地区增强内生发展能力。（国家乡村振兴局、国家发展改革委等按职责分工负责，5月底前出台相关政策，年内持续推进）
　　（十九）提高粮食和重要农产品供给保障能力。

　　**23．**加强种质资源保护利用和优良品种选育推广，开展农业关键核心技术攻关。（农业农村部、科技部牵头，国家林草局等按职责分工负责，9月底前出台相关政策，年内持续推进）提高高标准农田建设标准和质量，完善灌溉设施，强化耕地保护，坚决遏制耕地“非农化”、防止“非粮化”。推进农业机械化、智能化。（农业农村部、自然资源部等按职责分工负责，年内持续推进）建设国家粮食安全产业带。（国家发展改革委、农业农村部牵头，年内持续推进）建设农业现代化示范区。（农业农村部牵头，国家发展改革委、财政部、市场监管总局、国家乡村振兴局等按职责分工负责，年内持续推进）稳定种粮农民补贴，适度提高稻谷、小麦最低收购价，扩大完全成本和收入保险试点范围。（国家发展改革委、财政部、农业农村部、银保监会等按职责分工负责，12月底前完成）抓好农业生产。稳定粮食播种面积，提高单产和品质。多措并举扩大油料生产。发展畜禽水产养殖，稳定和发展生猪生产。加强动植物疫病防控。（农业农村部牵头，海关总署、国家林草局等按职责分工负责，年内持续推进）保障农产品市场供应和价格基本稳定。（国家发展改革委、农业农村部、财政部、商务部、国家粮食和储备局等按职责分工负责，年内持续推进）开展粮食节约行动。保障好14亿人的粮食安全。（国家发展改革委、农业农村部、财政部、国家粮食和储备局、商务部等按职责分工负责，年内持续推进）
　　（二十）扎实推进农村改革和乡村建设。

　　**24．**改善农村生产生活条件。巩固和完善农村基本经营制度，保持土地承包关系稳定并长久不变，稳步推进多种形式适度规模经营，加快发展专业化社会化服务。稳慎推进农村宅基地制度改革试点。发展新型农村集体经济。（农业农村部、国家乡村振兴局牵头，年内持续推进）深化供销社、集体林权、国有林区林场、农垦等改革。（国家发展改革委、自然资源部、农业农村部、国家林草局、供销合作总社等按职责分工负责，年内持续推进）提高土地出让收入用于农业农村比例。（农业农村部、国家发展改革委、财政部、自然资源部、住房城乡建设部等按职责分工负责，年内持续推进）强化农村基本公共服务和公共基础设施建设，促进县域内城乡融合发展。（国家发展改革委、农业农村部、国家乡村振兴局、财政部、住房城乡建设部、交通运输部、水利部、国家卫生健康委等按职责分工负责，年内持续推进）启动农村人居环境整治提升五年行动。（农业农村部、国家发展改革委、国家乡村振兴局、财政部、生态环境部、住房城乡建设部、文化和旅游部、国家卫生健康委等按职责分工负责，9月底前出台相关政策，年内持续推进）加强农村精神文明建设。（农业农村部、国家乡村振兴局、民政部、住房城乡建设部、文化和旅游部、广电总局等按职责分工负责，年内持续推进）保障农民工工资及时足额支付。（人力资源社会保障部牵头，年内持续推进）加快发展乡村产业，壮大县域经济，加强对返乡创业的支持，拓宽农民就业渠道。千方百计使亿万农民多增收、有奔头。（农业农村部、国家发展改革委、人力资源社会保障部、供销合作总社、共青团中央等按职责分工负责，年内持续推进）
　　七、实行高水平对外开放，促进外贸外资稳中提质
　　（二十一）推动进出口稳定发展。

　　**25．**加强对中小外贸企业信贷支持，扩大出口信用保险覆盖面、优化承保和理赔条件，深化贸易外汇收支便利化试点。（商务部、财政部、人民银行、银保监会、国家外汇局、进出口银行、中国出口信用保险公司等按职责分工负责，8月底前出台相关政策，年内持续推进）稳定加工贸易，发展跨境电商等新业态新模式，支持企业开拓多元化市场。发展边境贸易。创新发展服务贸易。（商务部牵头，6月底前出台相关政策，年内持续推进）优化调整进口税收政策，增加优质产品和服务进口。（财政部、国家发展改革委、商务部、海关总署、税务总局等按职责分工负责，4月底前出台相关政策，年内持续推进）加强贸易促进服务，办好进博会、广交会、服贸会及首届中国国际消费品博览会等重大展会。（商务部牵头，上海市、广东省、北京市、海南省人民政府等按职责分工负责，11月底前完成）推动国际物流畅通，清理规范口岸收费，不断提升通关便利化水平。（国家发展改革委、财政部、交通运输部、商务部、海关总署、国家邮政局、中国国家铁路集团有限公司等按职责分工负责，年内持续推进）
　　（二十二）积极有效利用外资。

　　**26．**进一步缩减外资准入负面清单。（国家发展改革委、商务部等按职责分工负责，10月底前完成）推动服务业有序开放，增设服务业扩大开放综合试点，制定跨境服务贸易负面清单。（商务部牵头，10月底前完成）推进海南自由贸易港建设，加强自贸试验区改革开放创新，推动海关特殊监管区域与自贸试验区统筹发展，发挥好各类开发区开放平台作用。（国家发展改革委、商务部、科技部、海关总署与海南省人民政府等按职责分工负责，8月底前出台相关政策，年内持续推进）促进内外资企业公平竞争，依法保护外资企业合法权益。欢迎外商扩大在华投资，分享中国开放的大市场和发展机遇。（国家发展改革委、商务部等按职责分工负责，年内持续推进）
　　（二十三）高质量共建“一带一路”。

　　**27．**坚持共商共建共享，坚持以企业为主体、遵循市场化原则，健全多元化投融资体系，强化法律服务保障，有序推动重大项目合作，推进基础设施互联互通。提升对外投资合作质量效益。（国家发展改革委、商务部、外交部牵头，有关部门按职责分工负责，年内持续推进）
　　（二十四）深化多双边和区域经济合作。

　　**28．**实施更大范围、更宽领域、更深层次对外开放，更好参与国际经济合作。（国家发展改革委、商务部牵头，年内持续推进）坚定维护多边贸易体制。推动区域全面经济伙伴关系协定尽早生效实施、中欧投资协定签署，加快中日韩自贸协定谈判进程，积极考虑加入全面与进步跨太平洋伙伴关系协定。（商务部牵头，年内持续推进）在相互尊重基础上，推动中美平等互利经贸关系向前发展。（国家发展改革委、财政部、农业农村部、商务部、人民银行、海关总署等按职责分工负责，年内持续推进）与世界各国扩大相互开放，实现互利共赢。（商务部牵头，年内持续推进）
　　八、加强污染防治和生态建设，持续改善环境质量
　　（二十五）继续加大生态环境治理力度。

　　**29．**深入实施可持续发展战略，巩固蓝天、碧水、净土保卫战成果，促进生产生活方式绿色转型。（国家发展改革委、生态环境部、住房城乡建设部等按职责分工负责，年内持续推进）强化大气污染综合治理和联防联控，加强细颗粒物和臭氧协同控制，北方地区清洁取暖率达到70%。（生态环境部、国家发展改革委、科技部、公安部、工业和信息化部、财政部、自然资源部、住房城乡建设部、交通运输部、农业农村部、市场监管总局、国家能源局、中国气象局等按职责分工负责，12月底前完成）整治入河入海排污口和城市黑臭水体，提高城镇生活污水收集和园区工业废水处置能力，严格土壤污染源头防控，加强农业面源污染治理。（生态环境部、国家发展改革委、财政部、住房城乡建设部、水利部、农业农村部等按职责分工负责，年内持续推进）继续严禁洋垃圾入境。有序推进城镇生活垃圾分类处置。推动快递包装绿色转型。加强危险废物医疗废物收集处理。（国家发展改革委、工业和信息化部、公安部、生态环境部、住房城乡建设部、商务部、国家卫生健康委、海关总署、市场监管总局、国家邮政局等按职责分工负责，年内持续推进）研究制定生态保护补偿条例。落实长江十年禁渔，实施生物多样性保护重大工程，科学推进荒漠化、石漠化、水土流失综合治理，持续开展大规模国土绿化行动，保护海洋生态环境，推进生态系统保护和修复，让我们生活的家园拥有更多碧水蓝天。（国家发展改革委、科技部、司法部、财政部、自然资源部、生态环境部、住房城乡建设部、交通运输部、农业农村部、水利部、国家林草局等按职责分工负责，年内持续推进）
　　（二十六）扎实做好碳达峰、碳中和各项工作。

　　**30．**制定2030年前碳排放达峰行动方案。优化产业结构和能源结构。推动煤炭清洁高效利用，大力发展新能源，在确保安全的前提下积极有序发展核电。扩大环境保护、节能节水等企业所得税优惠目录范围，促进新型节能环保技术、装备和产品研发应用，培育壮大节能环保产业，推动资源节约高效利用。加快建设全国用能权、碳排放权交易市场，完善能源消费双控制度。提升生态系统碳汇能力。以实际行动为全球应对气候变化作出应有贡献。（国家发展改革委、生态环境部、外交部、科技部、工业和信息化部、财政部、自然资源部、交通运输部、住房城乡建设部、人民银行、税务总局、市场监管总局、银保监会、国家能源局、国家国防科工局、国家林草局等按职责分工负责，年内持续推进）实施金融支持绿色低碳发展专项政策，设立碳减排支持工具。（人民银行牵头，生态环境部等按职责分工负责，6月底前出台相关政策，年内持续推进）
　　九、切实增进民生福祉，不断提高社会建设水平
　　（二十七）发展更加公平更高质量的教育。

　　**31．**构建德智体美劳全面培养的教育体系。推动义务教育优质均衡发展和城乡一体化，加快补齐农村办学条件短板。进一步提高学前教育入园率，完善普惠性学前教育保障机制，支持社会力量办园。鼓励高中阶段学校多样化发展，加强县域高中建设。增强职业教育适应性，深化产教融合、校企合作，深入实施职业技能等级证书制度。办好特殊教育、继续教育，支持和规范民办教育发展。分类建设一流大学和一流学科，加快优化学科专业结构，加强基础学科和前沿学科建设，促进新兴交叉学科发展。支持中西部高等教育发展。加大国家通用语言文字推广力度。发挥在线教育优势，完善终身学习体系。倡导全社会尊师重教。深化教育评价改革，健全学校家庭社会协同育人机制，规范校外培训。加强师德师风建设。在教育公平上迈出更大步伐，更好解决进城务工人员子女就学问题，高校招生继续加大对中西部和农村地区倾斜力度，努力让广大学生健康快乐成长，让每个孩子都有人生出彩的机会。（教育部牵头，国家发展改革委、科技部、工业和信息化部、民政部、财政部、人力资源社会保障部、市场监管总局、银保监会、国家卫生健康委、体育总局、国家民委、全国妇联、国务院妇儿工委办公室、农业农村部、中国残联等按职责分工负责，年内持续推进）健全教师工资保障长效机制，加大对乡村教师培训、在职提升学历、职称评定的政策倾斜，改善乡村教师待遇。（人力资源社会保障部、教育部牵头，财政部等按职责分工负责，年内持续推进）今年财政支出要更多向量大面广的民生领域倾斜，特别是义务教育和基本医疗。（财政部牵头，有关部门按职责分工负责，12月底前完成）
　　（二十八）推进卫生健康体系建设。

　　**32．**坚持预防为主，持续推进健康中国行动，深入开展爱国卫生运动。（国家卫生健康委牵头，国家发展改革委、教育部、生态环境部、住房城乡建设部、农业农村部、体育总局、国家中医药局等按职责分工负责，年内持续推进）深化疾病预防控制体系改革，强化基层公共卫生体系，创新医防协同机制，健全公共卫生应急处置和物资保障体系，建立稳定的公共卫生事业投入机制。加强精神卫生和心理健康服务。深化公立医院综合改革，扩大国家医学中心和区域医疗中心建设试点，加强全科医生和乡村医生队伍建设，提升县级医疗服务能力，加快建设分级诊疗体系。（国家卫生健康委、国家发展改革委、财政部、教育部、工业和信息化部、人力资源社会保障部、商务部、交通运输部、国务院国资委、国家粮食和储备局、国家中医药局、国家医保局、国家药监局等按职责分工负责，年内持续推进）坚持中西医并重，实施中医药振兴发展重大工程。（国家中医药局、国家卫生健康委、国家发展改革委牵头，教育部、科技部、财政部、农业农村部、国家医保局、国家药监局等按职责分工负责，年内持续推进）支持社会办医，促进“互联网＋医疗健康”规范发展。（国家卫生健康委、国家发展改革委、工业和信息化部、国家医保局、国家中医药局等按职责分工负责，年内持续推进）强化食品药品疫苗监管。（市场监管总局、国家药监局、海关总署、农业农村部、国家卫生健康委、工业和信息化部等按职责分工负责，年内持续推进）优化预约诊疗等便民措施，努力让大病、急难病患者尽早得到治疗。（国家卫生健康委、国家中医药局牵头，年内持续推进）要多措并举加大对县乡医院、卫生院的投入。（财政部、国家卫生健康委等按职责分工负责，年内持续推进）要采取一些让群众切实感受得到的措施，扩大基本医保门诊报销、常见病药品报销范围等。（国家医保局牵头，年内持续推进）

　　**33．**居民医保和基本公共卫生服务经费人均财政补助标准分别再增加30元和5元。（财政部、国家卫生健康委、国家医保局牵头，5月底前出台相关政策，年内持续推进）推动基本医保省级统筹。（国家医保局牵头，财政部、税务总局等按职责分工负责，年内持续推进）推动门诊费用跨省直接结算。（国家医保局牵头，年内持续推进）建立健全门诊共济保障机制，逐步将门诊费用纳入统筹基金报销。（国家医保局牵头，财政部等按职责分工负责，5月底前出台相关政策，年内持续推进）完善短缺药品保供稳价机制。（工业和信息化部、国家卫生健康委、国家医保局、市场监管总局等按职责分工负责，年内持续推进）采取把更多慢性病、常见病药品和高值医用耗材纳入集中带量采购等办法，进一步明显降低患者医药负担。（国家医保局牵头，年内持续推进）到明年年底前每个县至少要确定1家定点医疗机构，提供包括门诊费用在内的医疗费用跨省直接结算。（国家医保局牵头，2022年12月底前完成）
　　（二十九）保障好群众住房需求。

　　**34．**坚持房子是用来住的、不是用来炒的定位，稳地价、稳房价、稳预期。解决好大城市住房突出问题，通过增加土地供应、安排专项资金、集中建设等办法，切实增加保障性租赁住房和共有产权住房供给，规范发展长租房市场，降低租赁住房税费负担，尽最大努力帮助新市民、青年人等缓解住房困难。（住房城乡建设部牵头，国家发展改革委、财政部、自然资源部、人民银行、税务总局、银保监会等按职责分工负责，年内持续推进）
　　（三十）加强基本民生保障。

　　**35．**提高退休人员基本养老金。（人力资源社会保障部、财政部牵头，4月底前出台相关政策，年内持续推进）提高优抚对象抚恤和生活补助标准。（财政部、退役军人部等按职责分工负责，年内持续推进）推进基本养老保险全国统筹。（人力资源社会保障部、财政部牵头，税务总局等按职责分工负责，年内持续推进）规范发展第三支柱养老保险。（人力资源社会保障部、财政部、银保监会牵头，9月底前出台相关政策，年内持续推进）完善全国统一的社会保险公共服务平台。（人力资源社会保障部牵头，年内持续推进）加强军人军属、退役军人和其他优抚对象优待工作，健全退役军人工作体系和保障制度。（退役军人部牵头，有关部门按职责分工负责，年内持续推进）继续实施失业保险保障扩围政策。（人力资源社会保障部、财政部牵头，4月底前出台相关政策，年内持续推进）

　　**36．**促进医养康养相结合，稳步推进长期护理保险制度试点。（国家医保局、财政部、国家卫生健康委等按职责分工负责，年内持续推进）发展普惠型养老服务和互助性养老。发展婴幼儿照护服务。发展社区养老、托幼、用餐、保洁等多样化服务，加强配套设施和无障碍设施建设，实施更优惠政策，让社区生活更加便利。（民政部、国家发展改革委、财政部、人力资源社会保障部、自然资源部、住房城乡建设部、商务部、国家卫生健康委、市场监管总局、国家中医药局、全国老龄办、全国妇联、国务院妇儿工委办公室、中国残联等按职责分工负责，年内持续推进）完善传统服务保障措施，为老年人等群体提供更周全更贴心的服务。推进智能化服务要适应老年人、残疾人需求，并做到不让智能工具给他们日常生活造成障碍。（国家发展改革委、国家卫生健康委牵头，有关部门按职责分工负责，年内持续推进）健全帮扶残疾人、孤儿等社会福利制度，加强残疾预防，提升残疾康复服务质量。（民政部、中国残联牵头，国家发展改革委、财政部、教育部、公安部、人力资源社会保障部、住房城乡建设部、国家卫生健康委、国家医保局、共青团中央、全国妇联等按职责分工负责，年内持续推进）分层分类做好社会救助，及时帮扶受疫情灾情影响的困难群众，坚决兜住民生底线。（民政部牵头，有关部门按职责分工负责，年内持续推进）对兴办社区服务业要在税收等方面给予优惠支持，为家庭养老托幼减轻负担、送去温暖。（财政部、税务总局等按职责分工负责，年内持续推进）
　　（三十一）更好满足人民群众精神文化需求。

　　**37．**培育和践行社会主义核心价值观，弘扬伟大抗疫精神和脱贫攻坚精神，推进公民道德建设。繁荣新闻出版、广播影视、文学艺术、哲学社会科学和档案等事业。加强互联网内容建设和管理，发展积极健康的网络文化。传承弘扬中华优秀传统文化，加强文物保护利用和非物质文化遗产传承，建设国家文化公园。推进城乡公共文化服务体系一体建设，创新实施文化惠民工程，倡导全民阅读。（文化和旅游部、国家发展改革委、广电总局、教育部、国家文物局等按职责分工负责，年内持续推进）深化中外人文交流。（教育部等按职责分工负责，年内持续推进）完善全民健身公共服务体系。（体育总局、国家发展改革委、住房城乡建设部等按职责分工负责，年内持续推进）精心筹办北京冬奥会、冬残奥会等综合性体育赛事。（有关部门与北京市、河北省人民政府按职责分工负责，年内持续推进）
　　（三十二）加强和创新社会治理。

　　**38．**夯实基层社会治理基础，健全城乡社区治理和服务体系，推进市域社会治理现代化试点。（民政部、工业和信息化部、公安部、司法部、住房城乡建设部、农业农村部、国家卫生健康委、国家乡村振兴局等按职责分工负责，年内持续推进）加强社会信用体系建设。（国家发展改革委、人民银行牵头，有关部门按职责分工负责，年内持续推进）大力发展社会工作，支持社会组织、人道救助、志愿服务、公益慈善发展。保障妇女、儿童、老年人、残疾人合法权益。（民政部、国家发展改革委、财政部、教育部、公安部、人力资源社会保障部、住房城乡建设部、国家卫生健康委、国家医保局、国家乡村振兴局、国务院妇儿工委办公室、全国老龄办、全国妇联、中国残联、中国红十字会总会、共青团中央等按职责分工负责，年内持续推进）继续完善信访制度，推进矛盾纠纷多元化解。（国家信访局牵头，有关部门按职责分工负责，年内持续推进）加强法律援助工作，启动实施“八五”普法规划。（司法部、全国普法办牵头，年内持续推进）加强应急救援力量建设，提高防灾减灾抗灾救灾能力，切实做好洪涝干旱、森林草原火灾、地质灾害、地震等防御和气象服务。完善和落实安全生产责任制，深入开展安全生产专项整治三年行动，坚决遏制重特大事故发生。（应急部、工业和信息化部、公安部、自然资源部、生态环境部、住房城乡建设部、交通运输部、水利部、中国地震局、中国气象局、国家粮食和储备局、国家林草局、国家国防科工局等按职责分工负责，年内持续推进）完善社会治安防控体系，常态化开展扫黑除恶斗争，防范打击各类犯罪，维护社会稳定和安全。（公安部牵头，年内持续推进）
　　十、全面加强政府自身建设
　　（三十三）落实全面从严治党要求，加强法治政府、廉洁政府建设。

　　**39．**各级政府要增强“四个意识”、坚定“四个自信”、做到“两个维护”，自觉在思想上政治上行动上同以习近平同志为核心的党中央保持高度一致，践行以人民为中心的发展思想，不断提高政治判断力、政治领悟力、政治执行力，落实全面从严治党要求。扎实开展党史学习教育。加强法治政府建设，切实依法行政。坚持政务公开。严格规范公正文明执法。依法接受同级人大及其常委会的监督，自觉接受人民政协的民主监督，主动接受社会和舆论监督。强化审计监督。支持工会、共青团、妇联等群团组织更好发挥作用。深入推进党风廉政建设和反腐败斗争，锲而不舍落实中央八项规定精神。政府工作人员要自觉接受法律监督、监察监督和人民监督。加强廉洁政府建设，持续整治不正之风和腐败问题。必须立足社会主义初级阶段基本国情，着力办好自己的事。要始终把人民放在心中最高位置，坚持实事求是，求真务实谋发展、惠民生。要注重解民忧、纾民困，及时回应群众关切，持续改善人民生活。要力戒形式主义、官僚主义，切忌在工作中搞“一刀切”，切实为基层松绑减负。要居安思危，增强忧患意识，事不畏难、责不避险，有效防范化解各种风险隐患。要调动一切可以调动的积极因素，推进改革开放，更大激发市场主体活力和社会创造力，用发展的办法解决发展不平衡不充分问题。要担当作为，实干苦干，不断创造人民期待的发展业绩。（国务院各部门分别负责，年内持续推进）
　　十一、做好民族、宗教、侨务、国防工作
　　（三十四）全面贯彻党的民族政策、宗教政策和侨务政策。

　　**40．**坚持和完善民族区域自治制度，全面贯彻党的民族政策，铸牢中华民族共同体意识，促进各民族共同团结奋斗、共同繁荣发展。（国家民委牵头，有关部门按职责分工负责，年内持续推进）全面贯彻党的宗教工作基本方针，坚持我国宗教的中国化方向，积极引导宗教与社会主义社会相适应。全面贯彻党的侨务政策，维护海外侨胞和归侨侨眷合法权益，更大凝聚中华儿女共创辉煌的磅礴力量。（有关部门按职责分工负责，年内持续推进）
　　（三十五）大力支持国防和军队建设。

　　**41．**深入贯彻习近平强军思想，贯彻新时代军事战略方针，坚持党对人民军队的绝对领导，严格落实军委主席负责制，聚焦建军一百年奋斗目标，推进政治建军、改革强军、科技强军、人才强军、依法治军，加快机械化信息化智能化融合发展。全面加强练兵备战，统筹应对各方向各领域安全风险，提高捍卫国家主权、安全、发展利益的战略能力。完善国防动员体系，强化全民国防教育。（有关部门按职责分工负责，年内持续推进）优化国防科技工业布局。（国家国防科工局牵头，年内持续推进）各级政府要大力支持国防和军队建设，深入开展“双拥”活动，谱写鱼水情深的时代华章。（退役军人部牵头，年内持续推进）
　　十二、做好港澳台、外交工作
　　（三十六）全面准确贯彻“一国两制”、“港人治港”、“澳人治澳”、高度自治的方针。

　　**42．**完善特别行政区同[宪法](https://www.pkulaw.com/chl/7c7e81f43957c58bbdfb.html)和基本法实施相关的制度和机制，落实特别行政区维护国家安全的法律制度和执行机制。坚决防范和遏制外部势力干预港澳事务，支持港澳发展经济、改善民生，保持香港、澳门长期繁荣稳定。（国务院港澳办牵头，年内持续推进）
　　（三十七）坚持对台工作大政方针。

　　**43．**坚持一个中国原则和“九二共识”，推进两岸关系和平发展和祖国统一。高度警惕和坚决遏制“***”分裂活动。完善保障台湾同胞福祉和在大陆享受同等待遇的制度和政策，促进海峡两岸交流合作、融合发展，同心共创民族复兴美好未来。（有关部门按职责分工负责，年内持续推进）
　　（三十八）坚持独立自主的和平外交政策。

　　**44．**积极发展全球伙伴关系，推动构建新型国际关系和人类命运共同体。坚持开放合作，推动全球治理体系朝着更加公正合理的方向发展。持续深化国际和地区合作，积极参与重大传染病防控国际合作。同所有国家在相互尊重、平等互利基础上和平共处、共同发展，携手应对全球性挑战，为促进世界和平与繁荣不懈努力。（外交部、科技部、商务部、文化和旅游部、国家卫生健康委、海关总署、国际发展合作署、国家中医药局、国家药监局、中国红十字会总会等按职责分工负责，年内持续推进）
　　今年是我国现代化建设进程中具有特殊重要性的一年。我们要更加紧密地团结在以习近平同志为核心的党中央周围，高举中国特色社会主义伟大旗帜，以习近平新时代中国特色社会主义思想为指导，按照党中央、国务院决策部署，增强政治意识，提高政治站位，切实增强大局意识和系统观念，齐心协力，开拓进取，努力完成全年目标任务，确保兑现对人民的承诺。一是强化组织领导。各地区、各部门要精心组织，落实工作责任制，明确时间表、责任人和成果形式，把各项工作往前赶，全力以赴抓落实，把政府对人民的承诺变成促发展、惠民生的新成效。要锚定2035年远景目标，进一步细化实化“十四五”时期经济社会发展目标任务，统筹安排做好年度工作和“十四五”工作，确保取得实实在在的成效。有关地区和部门要对照分工意见抓紧制定重点工作分工实施方案，于4月6日前报国务院。二是密切协调联动。各地区、各部门要加强沟通协商，形成工作合力，对跨领域跨部门跨地区事项，要加强协同配合。各部门要加强对地方的指导服务，深入调研了解政策落实的进展情况、困难问题和成效经验，及时完善政策。各地区要加强与有关部门对接，结合当地实际情况，以钉钉子精神抓好政策措施落地落实落细。三是规范督查督办。各地区、各部门要加强督促检查和跟踪督办，有力有序推动主要目标任务如期完成。督查、督导等工作要规范进行，讲求实效，力戒形式主义、官僚主义，切实减轻基层负担。对作风漂浮、执行不力、效果不好的要加大督查和暗访力度，对有令不行、有禁不止、不作为、乱作为的要坚决追责问责。国务院办公厅要建立《政府工作报告》督查总台账，定期对账督办，重大情况及时报告。

国务院
　　2021年3月19日

© 北大法宝：（[www.pkulaw.com](https://www.pkulaw.com/)) 专业提供法律信息、法学知识和法律软件领域各类解决方案。北大法宝为您提供丰富的参考资料，正式引用法规条文时请与标准文本核对。
欢迎查看所有[产品和服务](https://www.pkulaw.com/helps/2.html)。法宝快讯：[如何快速找到您需要的检索结果？](https://www.pkulaw.com/helps/70.html)[法宝](https://www.pkulaw.com/helps/69.html)V6有何新特色？

扫描二维码阅读原文

**原文链接：**<https://www.pkulaw.com/chl/2f672261422acea0bdfb.html>
